# Photocatalytic and Antibacterial Properties of Ag-CuFe_2_O_4_@WO_3_ Magnetic Nanocomposite

**DOI:** 10.3390/nano11020298

**Published:** 2021-01-24

**Authors:** Mohammad Hossein Sayadi, Najmeh Ahmadpour, Shahin Homaeigohar

**Affiliations:** 1 Department of Environmental Engineering, Faculty of Natural Resources and Environment, University of Birjand, Birjand 9717434765, Iran; ahmadpour.najme@gmail.com; 2 Department of Environmental Engineering, Faculty of Agriculture and Natural Resources, Ardakan University, Ardakan 8951895491, Iran; 3 School of Science & Engineering, University of Dundee, Dundee DD1 4HN, UK

**Keywords:** photocatalyst, photodegradation, magnetic nanocomposite, antibacterial activity

## Abstract

This study aimed to synthesize a new magnetic photocatalytic nanosystem composed of Ag-CuFe_2_O_4_@WO_3_ and to investigate its photodegradation efficiency for two drug pollutants of Gemfibrozil (GEM) and Tamoxifen (TAM) under Ultraviolet (UV) light irradiation. In this regard, the effect of pH, catalyst dosage, and drug concentration was thoroughly determined. The largest photodegradation level for GEM (81%) and TAM (83%) was achieved at pH 5, a photocatalyst dosage of 0.2 g/L, drug concentration of 5 mg/L, and contact time of 150 min. The drug photodegradation process followed the pseudo first-order kinetic model. In addition to the photodegradation effect, the nanocomposites were proved to be efficient in terms of antibacterial activity, proportional to the Ag doping level. The Ag-CuFe_2_O_4_@WO_3_ nanocomposite exhibited a stable, efficient performance without an obvious catalytic loss after five successive cycles. Taken together, the developed magnetic photocatalyst is able to simultaneously disinfect wastewater streams and to degrade pharmaceutical contaminants and thus shows a promising potential for purification of multi-contaminant water systems.

## 1. Introduction

The pharmacological active compounds (PhACs) are an important, emerging group of organic contaminants that are persistent and show toxicity. They produce active metabolites when released into aquatic environments [1,2]. These compounds enter into the environment from the pharmaceutical industries, hospitals, and municipal wastewater treatment plants and after consumption in agriculture and aquaculture [3]. Numerous studies have been conducted on the formation and fate of PhACs’ active metabolites and their toxic effects. According to such investigations, these compounds are partially destroyed during conventional wastewater/water treatment processes and are non-biodegradable [4]. Although these compounds are present in the aqueous environment in trace quantities such as ng/L and mg/L, their chronic toxicity is challenging due to the steady exposure of human kind to these compounds. In fact, they are considered as pollutants with side effects on the aquatic organisms and account for a negative impact on human health [5]. Among various drug pollutants, Gemfibrozil (5-(2,5-Dimethylphenoxy)-2,2-dimethylpentanoic acid (GEM)), a fibrous hypolipidemic agent that is effective in reducing the incidence of coronary heart disease, and Tamoxifen 2-[4-[(Z)-1,2-diphenylbut-1-enyl]phenoxy]-*N*,*N*-dimethyl ethanamine (TAM), a nonsteroidal anti-estrogen drug that is widely used in the treatment and prevention of breast cancer, are frequently found in the wastewater streams [6]. Since conventional wastewater treatment processes are unable to remove these pharmaceutical compounds [7] and due to the fact that they are hardly biodegraded, they enter into surface water and groundwater. Such a contamination is considered as an environmental challenge and needs to be addressed through appropriate approaches. In recent years, Advanced Oxidation Processes (AOPs) have been widely used as effective methods for the removal of organic pollutants [8,9,10,11]. Among the various AOPs, photocatalysis has proved to be notably efficient in degradation of pharmaceuticals [12]. While semiconductor materials have shown a high potential for photodecomposition of different organic pollutants, commercial visible light photocatalysts are unstable or lose their efficiency upon irradiation. To overcome such limitations, group II–VI semiconductors whose energy gaps span the visible light spectral range have been suggested as superior, compatible substitutes [13,14]. Extensive agglomeration, difficult separation, and recovery are the other shortcomings that have hindered the large utility of photocatalytic nanoparticles on a scalable, industrial scale [14,15,16]. One excellent strategy to address the abovementioned limitations is hybridization of such photocatalytic nanoparticles with other supplementary, supportive components as a nanocomposite system.

As one of the newly studied photocatalysts, tungsten trioxide (WO_3_) has shown promising potentials such as a low band gap (2.6 eV), non-toxicity, low cost, light sensitivity, chemical stability, and environmental friendliness. Moreover, thanks to offering a photocatalytic activity under visible light, WO_3_ nanoparticles have been appealing for further research [13,17]. Despite such merits, recovery and separation of the WO_3_ nanoparticles from the reaction medium are challenging [18], and they also suffer from a relatively low specific surface area (2.7 m^2^/g) [19]. To overcome these bottlenecks, magnetic nanocomposites have been coupled with WO_3_ as a core–shell structure. Accordingly, not only the catalytic performance of WO_3_ is improved, but it can also be readily separated from water [20].

MFe_2_O_4_ (M = Zn, Ag, Cu, Ni, or Co) magnetic nanoparticles are thermally stable and show an optimum photocatalytic activity [21]. These magnetic nanoparticles are p-type and can bind to n-type semiconductors and form p-n catalytic nanocomposites such as ZnFe_2_O_4_/TiO_2_, CuFe_2_O_4_/TiO_2_, and CuFe_2_O_4_/WO_3_ [22]. As a result of such a combination, the electron-hole recombination rate is reduced and the photocatalytic efficiency is improved. As an example, a composite photocatalyst composed of CuFe_2_O_4_ plus other semiconductors such as WO_3_ can be easily activated by visible light. In addition, such a magnetic photocatalyst can readily enable recovery of WO_3_ [5]. These kinds of photocatalysts are also biocompatible and show a high potential for a wide range of applications in photocatalysis, CO_2_ reduction, energy conversion, solar energy production, and supercapacitors [23].

With respect to the other bottleneck of WO_3_ which is its low specific surface area, different metal nanoparticle-based coatings have been implemented. The presence of metal nanoparticles such as Au, Ag, Pt, or Pd on the surface of WO_3_, acting as electron receptors, prevents the recombination of electron-hole pairs and thus increases the surface charge transfer efficiency in the as-developed composites [24,25]. In addition, metal nanoparticles improve the visible light absorption based on surface plasmon resonance [26]. Specifically, Ag nanoparticles bring along additional merits such as antibacterial effects, as well. The inorganic antibacterial materials, particularly antibacterial metals such as Zn, Ag, Hg, and Cu, have been noticed owing to their superior properties over traditionally applied organic reagents in terms of thermal resistance, chemical stability, safety, and long term efficacy. Among them, Ag has the strongest antibacterial activity and has been extensively used as a bactericide since a long time ago due to its wide antibacterial spectrum, stability, and durability.

In this study, for the first time, we addressed the shortcomings of WO_3_ photocatalyst by coupling it with the magnetic phase of CuFe_2_O_4_, to ease its recovery, and Ag nanoparticles, to endow it with the additional feature of bactericidal activity. It is worth noting that such additional components may extend the applicability of the photocatalyst to under light irritation. Eventually, the as-synthesized nanocomposite was challenged for photodecomposition of two aforementioned organic (drug) pollutant models.

## 2. Materials and Methods

### 2.1. Materials

Iron (III) chloride, tungsten hexachloride (WCl_6_), polyethylene glycol (PEG), hydrochloric acid, copper (II) acetate (Cu(CH_3_COO)_2_), potassium ferricyanide (K_3_Fe(CN)_6_), polyvinyl pyrrolidone (PVP), and ethanol were purchased from Merck (Darmstadt, Germany). Silver nitrate, Tamoxifen, and Gemfibrozil were obtained from Sigma Aldrich (St. Louis, MO, USA). All the materials were used without further purification. Deionized water was used to prepare the aqueous solutions in this study.

### 2.2. Synthesis of the WO_3_ Nanoparticles

A solvothermal method was applied for synthesis of the WO_3_ nanoparticles. To do so, 1.5 mmol of WCl_6_ and 0.3 g of PEG were added to 70 mL of ethanol and then sonicated. Subsequently, the as-prepared solution was placed in an autoclave and heated at 110 °C for 3 h. After cooling down to the ambient temperature, the synthesized nanoparticles were collected and washed several times with deionized water and finally dried in an oven at 60 °C for 8 h [27].

### 2.3. Synthesis of the CuFe_2_O_4_@WO_3_ Nanoparticles

The CuF_e2_O_4_@WO_3_ nanoparticles were prepared via a chemical deposition method. First, 0.06 g of Cu(CH_3_COO)_2_·H_2_O was dissolved in 10 mL of deionized water. In parallel, 0.196 g of K_3_Fe(CN)_6_ was dissolved in 30 mL of deionized water. The latter solution was added to the first one drop-wise and stirred for 10 min within an ice bath. Afterwards, the WO_3_ nanoparticles were added to the mixture solution and stirred for one hour within an ice bath. The as-prepared suspension was later refrigerated at 0–4 °C for 120 min. Eventually, the resulting nanoparticles were collected and washed with distilled water and ethanol and dried in an oven at 60 °C for 3 h [28].

### 2.4. Synthesis of the Ag-CuFe_2_O_4_@WO_3_ Nanoparticles

At this step, 60 mg of CuFe_2_O_4_@WO_3_ was added to 15 mL of AgNO_3_ and vigorously stirred for 30 min. Then, 15 mL of ethanol containing 0.1 g of PVP was added to the suspension and stirred for 4 h at 60 °C. Finally, the mixture was transferred into a quartz tube and ultraviolet (UV) irradiated by a 150 W Hg lamp (λ = 365 nm) at the ambient temperature for several hours. At the end of the reaction, the as-synthesized nanoparticles were collected by a magnet, washed with distilled water and ethanol, and dried for 6 h at 70 °C [29].

### 2.5. Characterization of the Nanoparticles

Morphology of the nanoparticles was characterized using FESEM; Scanning Electron Microscope (TE-SCAN MIRA3 FESEM, Kohoutovice, Czech Republic). The crystalline structure of the Ag-CuFe_2_O_4_@WO_3_ nanoparticles was analyzed by X-ray Diffraction (XRD) (Rigaku MiniFlex 600, Tokyo, Japan) using Cu-Kα radiation (λ = 0.15418). The elemental analysis of the nanocomposite nanoparticles was performed using Electron Dispersion X-ray Spectroscopy. The Fourier Transforms Infrared Spectroscopy (FTIR) spectra of the nanoparticles were recorded in the scanning range of 400–4000 cm^−1^ (Shimadzu, FT-IR1650 spectrophotometer, Kyoto, Japan). Thermogravimetric Analysis (TGA) was performed by a Perkin Elmer instrument (Waltham, MA, USA) at the temperature range of 50 to 800 °C under nitrogen atmosphere. The magnetic properties of the nanoparticles were measured using a Vibrating Sample Magnetometer (VSM) (Lake Shore 7403, Westerville, OH, USA). The UV–Vis (DRS) spectra of the nanoparticles were recorded by a UV–Vis spectrometer (Shimadzu, UV-2550, Kyoto, Japan). X-ray Photoelectron Spectroscopy (XPS) was measured by a FOUNDRYMASTER Smart X-ray photoelectron device (Hitachi Advanced Technology, Tokyo, Japan) using monochromated Al-Kα as the X-ray source. Photoluminescence (PL) spectra of the nanoparticles were measured by using an excitation wavelength of 300 nm in an Agilent instrument (model G9800A, Santa Clara, CA, USA). Magnetic Electron Resonance (ESR) analysis was also done by a Bruker ELEXSYS 500 spectrometer (Billerica, MA, USA) to investigate the active free radicals.

### 2.6. Photodegradation Tests

Photodegradation levels of TAM and GEM by the Ag-CuFe_2_O_4_@WO_3_ magnetic nanoparticles were quantified in a photochemical reactor containing drug contaminated aqueous solutions. As the irradiation source, four UV lamps of 6 W (UVA light, 320–400 nm, with λ_max_ = 365 nm) were employed for the photocatalytic reactions. The photodegradation efficiency of the nanocomposite photocatalyst was determined at various pH levels (3, 5, 7, 9, and 11), catalyst dosages (0.05, 0.1, 0.2, 0.4, 0.6, and 0.8 g/L), and initial concentrations of TAM and GEM (5, 10, 20, 30, and 40 mg/L). Furthermore, the effect of H_2_O_2_ concentration (0.1, 0.2, 0.3, and 0.5 mg.L^-1^) and mineral salt ions (including Cl^−^, SO_4_^2−^, NO_3_^−^, and CO_3_^2−^ at the concentration of 1.5 mmol) on the photocatalytic degradation of TAM and GEM was investigated. An aeration pump (with the feed rate of 1.5 L/min) and a magnetic stirrer (with the rotation speed of 70 rpm) were used to mix and to provide the required oxygen in the measurements. NaOH and HCl (0.1 M) were used to adjust the pH of the tested solutions. It is worth noting that before switching on the lamps, the drug containing solutions were stirred for 30 min in the dark to ensure an adsorption/desorption equilibrium between the photocatalyst and the drug molecules. Then, at a specific time interval, 5 mL of the solution was taken from the reactor, the nanoparticles were separated using a magnetic field and analyzed by a spectrophotometer (at the wavelength of 236 and 276 nm for TAM and GEM, respectively). The photodegradation efficiency of the photocatalyst nanoparticles was determined through the following Equation (1) [30]:

(1) $$Removal\;\left(\%\right)=(\frac{C_{t}-C_{0}}{C_{t}})\times 100$$

### 2.7. Antibacterial Efficiency Tests

The antibacterial activity of Ag-CuFe_2_O_4_@WO_3_ nanoparticles against the *Escherichia coli* bacteria was determined via the Luria-Bertani (LB) counting method (the plate counting method). To differentiate the antibacterial effect of CuFe_2_O_4_@WO_3_ from that of Ag, this experiment was solely performed under the visible light. Accordingly, the achieved antibacterial activity is only attributed to the Ag nanoparticles. The antibacterial tests were conducted in a 100 mL sterilized glass container containing *E. coli* suspension (7 mL phosphate buffer solution containing the bacteria) and 0.4 g/L of the photocatalyst nanoparticles. The pH of the medium was kept neutral and it was incubated at 37 °C under magnetic stirring for up to 24 h. Then, 0.5 mL of the solutions was cultured on an agar plate and incubated at 37 °C for 16 h. Eventually, the number of isolated colonies was counted to represent the density of the remaining bacteria. As a control, the antibacterial efficiency of the CuFe_2_O_4_@WO_3_ nanoparticles was also measured to enable a comparison with the nanoparticles containing Ag. The antibacterial efficiency (*R*) was quantified via the Equation (2) [31]:

(2) $$R\left(\%\right)=\frac{A-B}{A}\times 100$$

where *R* is the bacterial reduction ratio, *A* is the number of bacterial colonies of the solution containing CuFe_2_O_4_@WO_3_, and *B* is the number of bacterial colonies of the solution containing Ag-CuFe_2_O_4_@WO_3_ after different isothermal incubation periods.

## 3. Results and Discussion

### 3.1. Morphology and Size of the Nanoparticles

**Figure 1:**
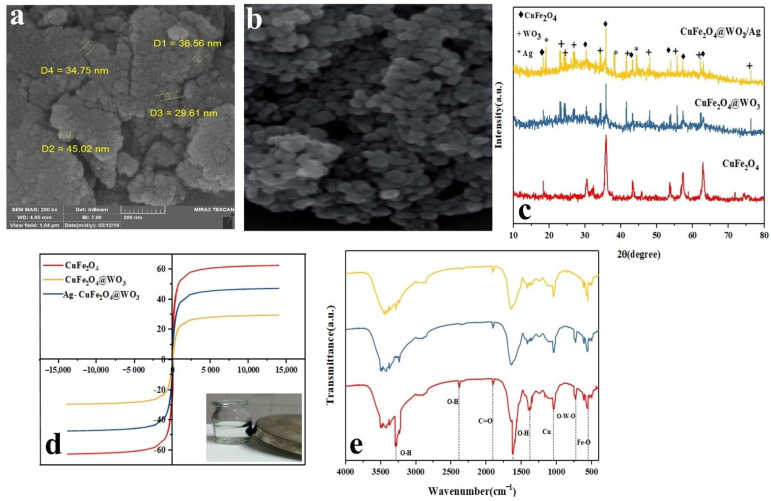
(**a**,**b**) FESEM images of the Ag-CuFe_2_O_4_@WO_3_ nanoparticles at different magnifications (the scale bar in (**a**) represents 200 nm); (**c**) XRD spectra for the Ag-CuFe_2_O_4_@WO_3_ nanoparticles; (**d**) magnetic behavior of the Ag-CuFe_2_O_4_@WO_3_ nanoparticles; (**e**) FTIR spectra of the Ag-CuFe_2_O_4_@WO_3_ nanoparticles representing their various bonds and functional groups.

The FESEM images of the Ag-CuFe_2_O_4_@WO_3_ nanoparticles are shown in Figure 1a,b. As seen in the images, the nanoparticles are almost uniform in terms of size and shape (spherical). The average diameter of the nanoparticles lies within the range of 30–45 nm. As a fact, the nanoparticles are agglomerated, due to their high magnetic properties, engendering their mutual attraction [3].

### 3.2. Crystallinity of the Nanoparticles

The crystalline structure of the CuFe_2_O_4_, CuFe_2_O_4_@WO_3_, and Ag-CuFe_2_O_4_@WO_3_ nanoparticles was evaluated via XRD analysis. As shown in Figure 1c, the diffraction peaks appearing at 2θ of 18.35°, 18.30°, 35.5°, 43.2°, 53.58°, 57.14°, and 62.74° are attributed to CuFe_2_O_4_ and the crystallographic planes of (111), (220), (311), (400), (422), (511), and (440), respectively (according to JCPDS no. 77-0010) [28]. On the other hand, the characteristic peaks seen at 2θ of 23°, 24.15°, 26.7°, 34°, 52.41°, 48°, 55.7°, 62.25°, and 23.76° correspond with WO_3_’s crystallographic planes of (001), (200), (120), (220), (221), (240), (132), and (422), respectively (JCPDS card no. 32-1395) [32]. For the composite structures, the lower dispersion radiation intensity of CuFe_2_O_4_ was caused by the WO_3_ coating and due to absorption of X-ray. The XRD spectrum of Ag-CuFe_2_O_4_@WO_3_ nanoparticles shows several additional peaks appearing at 19.04°, 38.11°, and 44.32° related to Ag component’s crystallographic planes of (110), (111), and (220), respectively (JCPDS code.00-001-1281). By coating WO_3_ nanoparticles, in addition to creating new peaks, the intensity of peaks in CuFe_2_O_4_@WO_3_ is also reduced. In other words, the decrease in the scattering intensity of CuFe_2_O_4_ as a result of WO_3_ coating is due to the absorption of X-rays through WO_3_.

### 3.3. Elemental Analysis of the Nanoparticles

The EDS analysis verified the presence of elements such as O, Fe, W, Ag, and Cu in the Ag-CuFe_2_O_4_@WO_3_ nanoparticles. The weight percentage of the elements is 11.5 (Cu), 24.1 (Fe), 33 (W), 22.3 (O), and 8.6 (Ag) (note that the numbers have been rounded). Among the five elements (O, Fe, W, Cu, and Ag) demonstrated, the W content is higher than that of CuFe_2_O_4_ which could be due to the formation of a WO_3_ layer on the CuFe_2_O_4_ nanoparticles. Furthermore, the signal of Ag around 2.78 keV confirms the presence of Ag nanoparticles in the nanocomposite.

### 3.4. Magnetic Properties of the Nanoparticles

The magnetic properties of CuFe_2_O_4_, CuFe_2_O_4_@WO_3_, and Ag-CuFe_2_O_4_@WO_3_ nanoparticles were investigated using a vibrating sample magnetometer at the ambient temperature. As shown in Figure 1d, the magnetic saturation of the CuFe_2_O_4_ nanoparticles was as large as 62.57 emu/g, indicating that these nanoparticles are supermagnetic, and their magnetic hysteresis loop passes through the origin of the coordinates [17]. The magnetic saturation of the nanocomposite nanoparticles of CuFe_2_O_4_@WO_3_ and Ag-CuFe_2_O_4_@WO_3_ was recorded as 47.32 emu/g and 29.49 emu/g, respectively, implying that in the presence of the coating, this property declines. However, paramagnetic properties of the nanoparticles are preserved and this allows easy separation of them from the solution under an external magnetic field [22].

### 3.5. Surface Chemistry of the Nanoparticles

The FTIR spectra the CuFe_2_O_4_, CuFe_2_O_4_@WO_3_, and Ag-CuFe_2_O_4_@WO_3_ nanoparticles are shown in Figure 1e. The band centered at 415 cm^−1^ and 1044 cm^−1^ are assigned to the stretching vibration of Fe–O and Cu, respectively, associated with copper ferrite [33,34]. The dips appearing at 3400 cm^−1^ and 2378 cm^−1^ relate to the stretching vibrations of O-H [35] and the one emerging at 2853 cm^−1^ corresponds to C=O vibration [36]. With respect to the WO_3_ phase, the bond stretching of O-W-O appears in the range of 600–800 cm^−1^ [37]. Lastly, incorporation of Ag into the CuFe_2_O_4_@WO_3_ nanoparticles leads to emergence of a new dip at 1383 cm^−1^, representing the Ag-O bond [38]. After the silver coating, there was no change in the position of the CuFe_2_O_4_@WO_3_ peaks, and only one vibrational tensile peak was created in 2004 (cm^−1^), which indicates the Ag-O bond. The peaks have not changed, indicating that WO_3_ and Ag have not destroyed the structure of CuFe_2_O_4_. Moreover, no notable peak shift implies that the components are merely physically connected and no significant hydrogen bonding or chelation has not taken place. FTIR spectra could clearly indicate the respective functional groups of each component in the structure of nanocomposites. This could also point out to preservation of each phase after production cycle and assures that the system benefits from unique function of each component without any notable compromise that could be resulted from intermolecular bonding.

### 3.6. Surface Chemical Composition of the Nanoparticles

**Figure 2:**
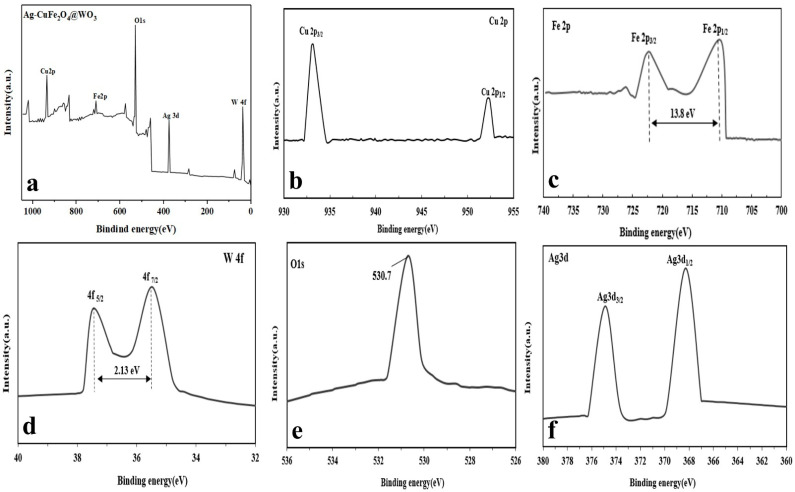
XPS analysis of the Ag-CuFe_2_O_4_@WO_3_ nanoparticles: (**a**) general XPS spectrum of the compound, and (**b**) Cu2p; (**c**) Fe2p; (**d**) W4f, (**e**) O1s; and (**f**) Ag3d spectrum of the compound.

Based on the XPS analysis (general spectrum), surface composition of the Ag-CuFe_2_O_4_@WO_3_ nanoparticles consists of Fe, Cu, W, Ag, and O atoms (Figure 2a). More precisely monitoring, Figure 2b shows that the Cu binding energy is represented in the two peaks of 933 and 953 eV, which are attributed to Cu 2p_3/2_ and Cu 2p_1/2_, respectively. There are two distinct peaks in the Fe 2p spectrum, appearing at 710.7 and 724.5 eV, corresponding to the binding energies of Fe 2p_1/2_ and Fe 2p_3/2_, respectively [39] (Figure 2c). The mentioned binding energies arise from CuFe_2_O_4_. After coating CuFe_2_O_4_ with WO_3_, as shown in Figure 2d, two new signals appear at 35.47 and 37.6 eV corresponding to W4f_7/2_ and W4f_5/2_, respectively. The peaks are separated with a gap of 2.13 eV [40]. Figure 2e shows the O1s spectrum of the nanoparticles with a binding energy peak at 530.7 eV, attributed to oxygen at the W-O bond [41]. The Ag3d surface spectrum of the nanoparticles possesses two peaks with the binding energies of 368.3 eV and 374.10 eV, as shown in Figure 2f, that are attributed to Ag3d_5/2_ and Ag3d_3/2_, respectively. The 5.8 eV gap between the two peaks indicates the presence of Ag. Noteworthy, with no track of Ag^+^, it can be confidently said that Ag ions have been reduced to metallic silver in the nanocomposite particles [5]. 

### 3.7. Band Gap Energy and Optical Activity of the Nanoparticles

**Figure 3:**
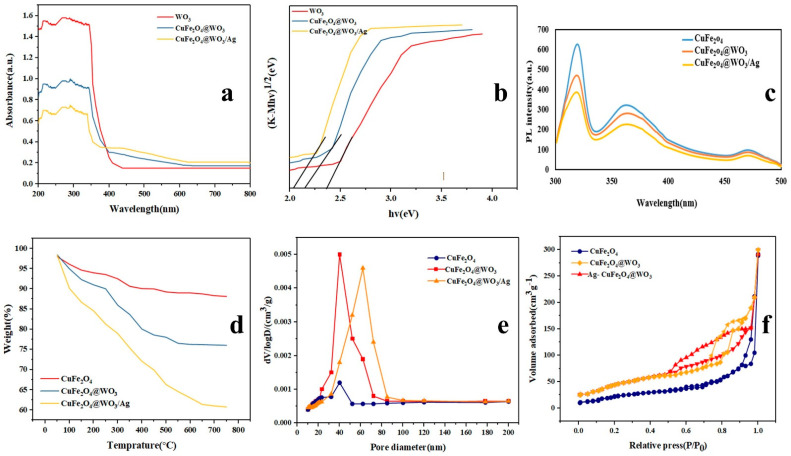
(**a**) UV–Vis DR spectra and (**b**) band gap energy curves of the Ag-CuFe_2_O_4_@WO_3_ nanoparticles compared to those of the WO_3_ and CuFe_2_O_4_@WO_3_ nanoparticles; (**c**) photoluminescence (PL) analysis of the Ag-CuFe_2_O_4_@WO_3_ nanoparticles; (**d**) TGA curve for the Ag-CuFe_2_O_4_@WO_3_ nanoparticles in comparison with the controls; (**e**) the pore diameter distribution curves and (**f**) N_2_ adsorption/desorption isotherms of the CuFe_2_O_4_, CuFe_2_O_4_@WO_3_, and Ag-CuFe_2_O_4_@WO_3_ nanoparticles.

The UV–Vis-diffuse reflectance spectra of the CuFe_2_O_4_, CuFe_2_O_4_@WO_3_, and Ag-CuFe_2_O_4_@WO_3_ nanoparticles are shown in Figure 3a. As seen here, the latter class of the nanoparticles containing Ag is able to absorb UV light and partly visible light and their light absorption intensity is higher than that for the CuFe_2_O_4_@WO_3_ nanoparticles. It is worth noting that the WO_3_ nanoparticles can only absorb UV radiation due to the wide band gap (2.42 eV) of WO_3_. In contrast, CuFe_2_O_4_ shows a narrow band gap and might cause a defect in the WO_3_ network and extend its optical activity to the visible light range. Thus, compared with WO_3_, the CuFe_2_O_4_@WO_3_ and Ag-CuFe_2_O_4_@WO_3_ photocatalysts can potentially offer a higher photodegradation efficiency under UV–Vis light irradiation. The band gap energy of the nanoparticles is equal to (1/2)(*Ahv*) where *hv* is the photon energy [42]. The band gap energy values of WO_3_, CuFe_2_O_4_@WO_3_, and Ag-CuFe_2_O_4_@WO_3_ nanoparticles are calculated as 2.42, 2.21, and 2.13 eV, respectively. Therefore, these kinds of nanoparticles exhibit a high photocatalytic activity under visible light irradiation. As shown in Figure 3b, the band gap energy in WO_3_ nanoparticles was 2.42 eV. When coated on CuFe_2_O_4_, the band gap energy in CuFe_2_O_4_@WO_3_ declines to 2.21 eV [43]. Furthermore, inclusion of Ag leads to further decrease of the band gap energy to 2.13 eV. The red shift of the optical response for the Ag-CuFe_2_O_4_@WO_3_ nanoparticles stems from interference of the semiconductor band gaps as well as the surface plasma resonance (SPR) effect of the spatially confined electrons in metallic Ag nanoparticles.

CuFe_2_O_4_ nanoparticles can absorb UV light and visible light due to their narrow band gap, thus increasing electron-hole recombination. After WO_3_ deposition, the adsorption capacity of CuFe_2_O_4_@WO_3_ in the visible light increases due to the synergistic effects of WO_3_ (transfer) and CuFe_2_O_4_ (adsorption). The Ag coating significantly increases the ability to absorb visible light, which is due to the plasmon resonance effect of the Ag surface. Therefore, with the coating of nanoparticles, the amount of band gap is reduced, thus it has the capability to absorb light and increase photocatalytic activity.

### 3.8. Electron Transfer Ability of the Nanoparticles

Photoluminescence (PL) spectroscopy was applied to determine the electron transfer ability of the photocatalytic nanoparticles and their capacity for separation of light-generated charge carriers. The PL spectra of the CuFe_2_O_4_, CuFe_2_O_4_@WO_3_, and Ag-CuFe_2_O_4_@WO_3_ nanoparticles are shown in Figure 3c. The main peak appearing at 320 nm represents the electron-hole recombination in the conduction and valence band of the photocatalyst. According to the PL spectra, the CuFe_2_O_4_ nanoparticles exhibit the highest PL emission intensity, implying their largest electron-hole recombination rate. However, upon coating of the CuFe_2_O_4_ core with WO_3_ and Ag, the peak intensity declines, indicating a decrease in the recombination rate of optical charge carriers. Such a lower electron-hole recombination rate can be interpreted as a higher photocatalytic activity and larger photodegradation efficiency of the photocatalyst nanoparticle.

### 3.9. Thermal Stability of the Nanoparticles

TGA curves of the CuFe_2_O_4_, CuFe_2_O_4_@WO_3_, and Ag-CuFe_2_O_4_@WO_3_ nanoparticles are shown in Figure 3d. Based on these curves, a weight loss of 17.2% takes place for the CuFe_2_O_4_ nanoparticles within the temperature range of 50–500 °C. Over 500 °C, no further weight loss occurs, indicating thermal stability of the CuFe_2_O_4_ nanoparticles at high temperatures. In the case of the CuFe_2_O_4_@WO_3_ nanoparticles, weight loss takes place in three steps. The initial weight loss occurs at 50–150 °C, due to evaporation of alcohol and absorbed water. The second weight loss is recorded between 250 °C and 500 °C, due to decomposition of some organic molecules [22]. The last weight loss happens in the temperature range of 500–800 °C. The Ag-CuFe_2_O_4_@WO_3_ nanoparticles show the largest weight loss due to water evaporation, phase transformation, decomposition of some residual organic molecules from solvent and/or removal of the hydroxyl groups present on the surface [44]. Thermal stability analysis of nanoparticles showed that with the addition of WO_3_ and Ag, thermal stability increases. This raised stability is due to the fact that the decomposition of organic matter in the reaction is reduced. Therefore, it adds to the stability of the nanoparticles and stabilizes the sample at 500 °C.

### 3.10. Surface Porosity of the Nanoparticles

Figure 3e,f show the surface pore diameter distribution and the N_2_ adsorption/desorption isotherms of the CuFe_2_O_4_, CuFe_2_O_4_@WO_3_, and Ag-CuFe_2_O_4_@WO_3_ nanoparticles, respectively. Regarding the CuFe_2_O_4_ nanoparticles, the average pore diameter was measured to be ~5 nm. The surface area and the total pore volume of these nanoparticles was 46.034 m^2^ g^−1^ and 0.457 cm^3^ g^−1^, respectively. The N_2_ adsorption/desorption isotherm of the CuFe_2_O_4_ is a type III isotherm according to IUPAC, implying insignificant surface porosity of the CuFe_2_O_4_ nanoparticles. When the CuFe_2_O_4_ core is coated with WO_3_ and further with Ag, the isotherms transform to the IUPAC IV isotherm with H_2_ hysteresis loops, witnessing the mesoporosity of the nanoparticles. The average pore diameter of the CuFe_2_O_4_@WO_3_ nanoparticles was equivalent to ~9 nm, and the surface area and total pore volume were determined to be 349.38 m^2^ g^−1^ and 0.449 cm^3^ g^−1^, respectively. Upon inclusion of Ag into the nanoparticles, the surface area raised up to 131.15 m^2^ g^−1^ and the average pore diameter and the total pore volume decreased to ~7 nm and 0.398 cm^3^ g^−1^, respectively.

### 3.11. Effect of Adsorption and Photolysis

**Figure 4:**
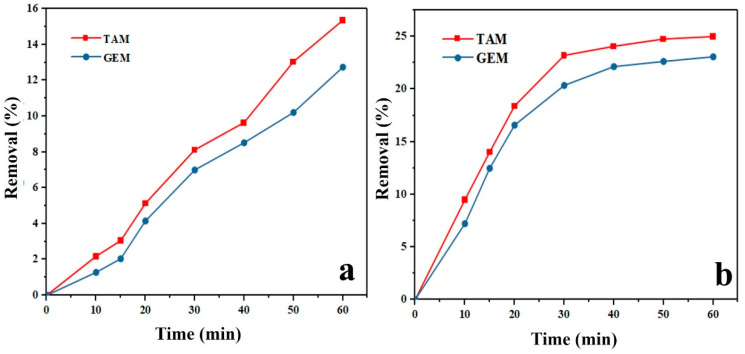
Photolysis (**a**) and adsorption (**b**) of Tamoxifen (TAM) and Gemfibrozil (GEM) by the Ag-CuFe_2_O_4_@WO_3_ nanoparticles. The photolysis conditions included: pH 7, 10 mg/L initial concentration of TAM and GEM, UV light irradiation for 60 min.

Photolysis trials were conducted in the absence of the photocatalyst, at pH 7, with 10 mg/L initial concentration of contaminants and under UV light irradiation for 60 min. The adsorption amount of Ag-CuFe_2_O_4_@WO_3_ nanocomposite at different times, *q_t_* (mg/g), was calculated using the following equation:

(3) $$q_{t}=\left(\left(C_{0}-C_{t}\right)V\right)/m,$$

where *C*_0_ was the initial concentration and *C_t_* concentration of the contaminant at time *t* (mg/L), *V* was the volume of the TAM and GEM solution, and *m* was the mass of Ag-CuFe_2_O_4_@WO_3_ (mg). Moreover, the adsorption capacity of Ag-CuFe_2_O_4_@WO_3_, *q_e_* (mg/g), was obtained according to the following equation:

(4) $$q_{e}=\left(\left(C_{0}-C_{e}\right)V\right)/m,$$

where *C*_0_ and *C_e_* were the initial concentration and pollutant equilibrium concentration (mg/L), respectively, *V* the volume of the TAM and GEM solutions (mL), and *m* the mass of Ag-CuFe_2_O_4_@WO_3_ (mg). As Figure 4a,b shows, the removal percentage of TAM and GEM contaminants under photolysis and adsorption was insignificant ( less than 30%).

### 3.12. Photodegradation Efficiency

**Figure 5:**
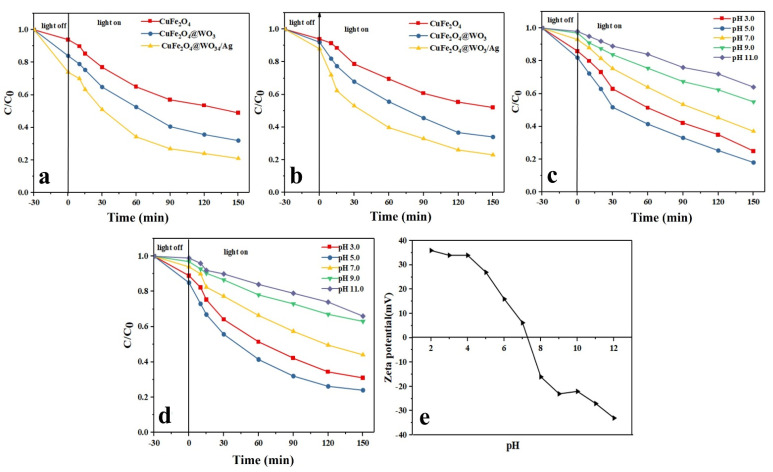
Photodegradation efficiency of the Ag-CuFe_2_O_4_@WO_3_ nanoparticles over a 150 min time period for TAM (**a**) and GEM; (**b**) effect of pH on the photocatalytic degradation of (**c**) TAM and (**d**) GEM; (**e**) Zeta potential measurement at different pHs for the Ag-CuFe_2_O_4_@WO_3_ nanoparticles.

The drug photodegradation behavior of the CuFe_2_O_4_, CuFe_2_O_4_@WO_3_, and Ag-CuFe_2_O_4_@WO_3_ nanoparticles was evaluated at pH 7, catalyst dosage of 0.1 g/L, and drug concentration of 10 mg/L. The results are shown in Figure 5a,b for TAM and GEM, respectively. In the absence of the nanoparticles, only a minor percentage of TAM and GEM was degraded under light irradiation. In contrast, upon inclusion of the photocatalysts, photodegradation efficiency increased and the Ag-CuFe_2_O_4_@WO_3_ nanoparticles showed the highest efficiency, followed by the CuFe_2_O_4_@WO_3_ nanoparticles. With addition of WO_3_, the electron-hole recombination declines, thus resulting in a more optimum photocatalytic performance. This efficiency further rises by addition of Ag nanoparticles, due to the lower band gap energy of Ag than the transfer band of WO_3_, leading to better charge carrier separation and less electron-hole recombination.

#### 3.12.1. Effect of pH

Given the crucial effect of pH on the surface charge of the adsorbents and the pollutant molecules, the influence of this parameter on removal of the drug models was monitored. In this regard, the photodegradation tests were conducted under different pH conditions between pH 3 and pH 11. As shown in Figure 5c,d, the highest photodegradation efficiency was achieved at pH 5, which is below the isoelectric point of the CuFe_2_O_4_ nanoparticles, i.e., 6.2 (Figure 5e). Under this pH level, the nanoparticles’ surface is protonated and positively charged and attracts the anionic molecules of the drugs. While over pH 6.2, an opposite situation is the case and the drug molecules are repelled from the negatively charged surface of the nanoparticles and therefore the degradation efficiency declines. pH can also affect the extent of production of the oxidative radicals [45]. Over pH 6.2, the photodegradation efficiency decreases due to a lower density of the hydroxyl radicals, thus a less oxidation potential [46]. Another reason for the lower photodegradation efficiency under alkaline conditions is the formation of insoluble compounds in the media, which hampers light penetration and thereby leads to a reduction in generation of the hydroxyl radicals [47,48].

#### 3.12.2. Effect of Initial Drug Concentration

**Figure 6:**
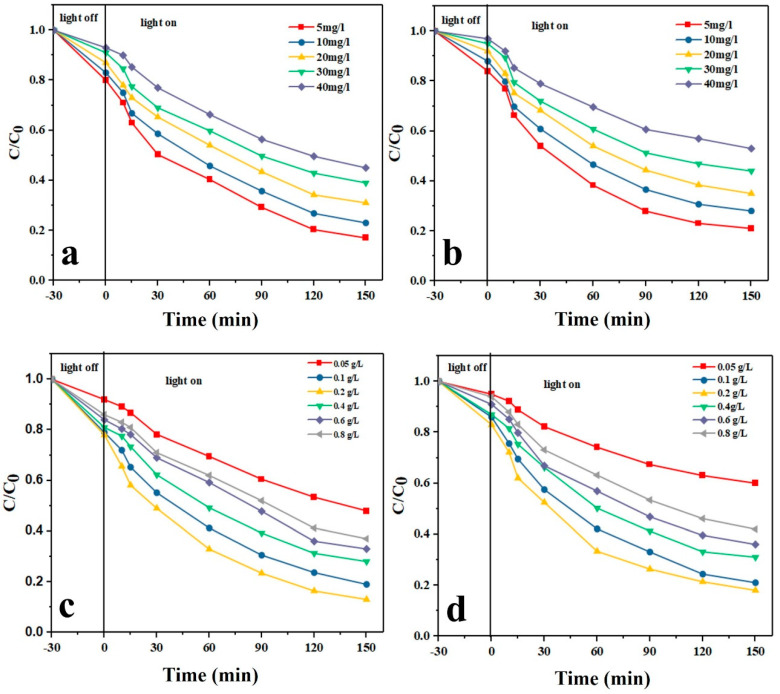
Effect of initial drug concentration on the photocatalytic degradation of (**a**) TAM and (**b**) GEM; effect of the photocatalyst dosage on the photocatalytic degradation of (**c**) TAM and (**d**) GEM.

As shown in Figure 6a,b, with increasing the initial drug concentration, the photodegradation efficiency for both drug models decreases. Another observation is that with increasing the radiation time, the photodegradation rate increases. The highest photodegradation efficiency was achieved at 5 mg/L drug concentration and after 150 min. Over a large time, the number of holes and hydroxyl radicals rises and this leads to an increased photocatalytic degradation efficiency [6,49]. In contrast, at higher drug concentrations, given the fixed amount of the catalyst, less hydroxyl radicals are available to interact and thus a lower photodegradation efficiency is obtained. Moreover, at higher drug concentrations, larger amounts of intermediate compounds are produced that can consume the present free radicals, thereby reducing the photodegradation efficiency [50]. The photolysis tests also implied that in the absence of the photocatalyst nanoparticles, the drugs are negligibly photodegraded solely due to their hydrolysis [51].

#### 3.12.3. Effect of the Photocatalyst Dosage

As shown in Figure 6c,d, with increasing the dosage of the Ag-CuFe_2_O_4_@WO_3_ photocatalyst up to 0.2 g/L, the photodegradation level of TAM and GEM rises, whereas the photodegradation rate declines. The results can be explained by the number of available active sites on the photocatalyst surface and the penetration of UV light into the drug aqueous solution. By increasing the photocatalyst dosage, the number of active sites and thereby the production level of hydroxyl radicals rises, which leads to a higher photodegradation efficiency [52]. On the other hand, when the photocatalyst dosage exceeds the optimal limit, i.e., 0.2 g/L, the solution gets turbid and the UV light penetration is hampered and the photodegradation efficiency declines [53].

#### 3.12.4. Photodegradation Kinetics

**Figure 7:**
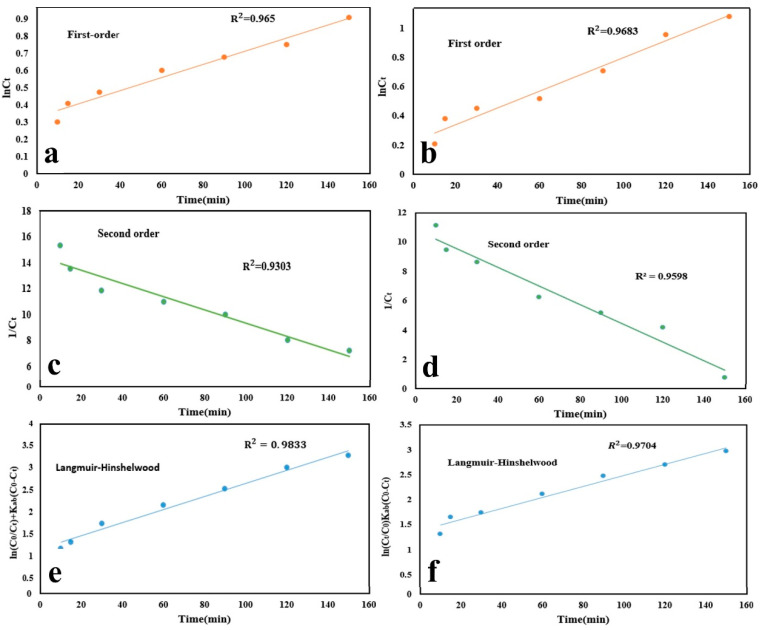
Kinetic plots of photodegradation process of (**a**,**c**,**e**) TAM and (**b**,**d**,**f**) GEM, obtained through several kinetic models of first-order, second-order, and Langmuir–Hinshelwood.

The kinetics of the photodegradation reactions was assessed to understand the underlying mechanisms (Figure 7a-f). For this purpose, optimum conditions for the photodegradation process of the drug models including pH 5, the initial drug concentration of 5–40 mg/L, and the photocatalyst dosage of 0.2 g/L were taken into account. Three possible kinetic models, including first-order kinetic model, second-order kinetic model, and Langmuir–Hinshelwood kinetic model were investigated [54].

First-order kinetic model

(5) $$InC_{t}=-K_{1}t+lnC_{0};$$

Second-order kinetic model

(6) $$1/C_{t}=-K_{2}t+1/C_{0};$$

Langmuir–Hinshelwood kinetics model

(7) $$ln\left(C_{t}/C_{0}\right)+\left(C_{0}-C_{t}\right)=-K_{3}K_{ab}t;$$

where *C*_0_ and *C_t_* represent the concentrations of TAM and GEM at time = 0 and time = *t*, respectively. *K*_1_, *K*_2_, and *K*_3_ are the rate constants of first-order, second-order, and Langmuir–Hinshelwood, respectively. In addition, *K_ab_* was exhibited as the Langmuir constant.

As shown in Figure 7a–f, study of TAM and GEM photocatalytic degradation kinetics using Ag-CuFe_2_O_4_@WO_3_ photocatalyst with these three kinetic models showed that the highest correlation coefficient was related to the Langmuir–Hinshelwood kinetic model, with the highest R^2^ of 0.9833 and 0.9704 for TAM and GEM, respectively [55,56].

#### 3.12.5. Effect of H_2_O_2_ Dosage

**Figure 8:**
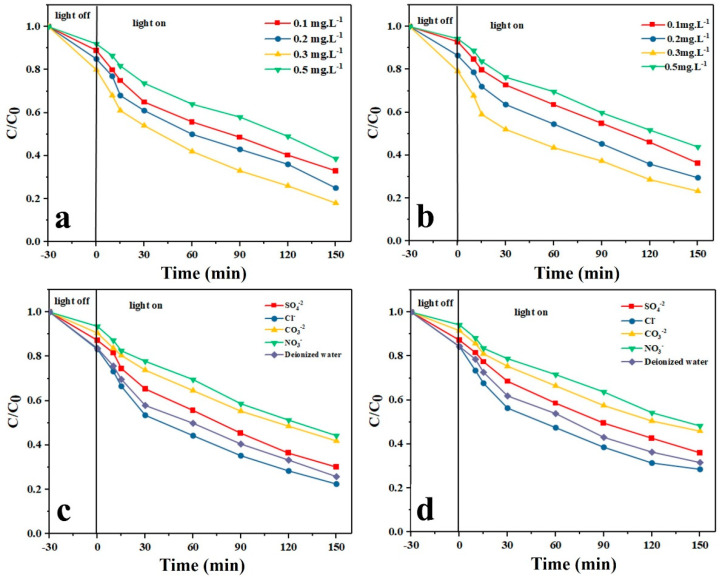
Effect of H_2_O_2_ dosage on the photocatalytic degradation of (**a**) TAM and (**b**) GEM; effect of anions on the photocatalytic degradation of (**c**) TAM and (**d**) GEM.

Figure 8a,b shows the effect of H_2_O_2_ dosage on the photodegradation of TAM and GEM drugs, respectively, by the Ag-CuFe_2_O_4_@WO_3_ nanoparticles. In these experiments, H_2_O_2_ in different dosages including 0.1, 0.2, 0.3, and 0.5 mg/L was added to the drug containing aqueous solutions and the photodegradation tests were performed under optimal conditions (photocatalyst dosage: 0.2 g/L, drug concentration: 5 mg/L, and pH 5). As shown in Figure 8 a,b, with increasing the dosage of H_2_O_2_, up to 0.5 mg/L, the photodegradation efficiency rises and thereafter it declines. The improved photodegradation efficiency could be attributed to the raised concentration of oxygen due to decomposition of H_2_O_2_ and also the contributing role of H_2_O_2_ in production of oxidative hydroxyl radicals. However, when H_2_O_2_ dosage exceeds a threshold, it starts to compete with the drug pollutants for reaction (consumption) with (of) the hydroxyl radicals, leading to generation of weaker radicals of ^•^OOH, which have a negligible impact on photodegradation of the drug molecules [57]. The influence of H_2_O_2_ dosage on the degradation of pollutants can be explained in terms of the number of generated OH radicals and the capture of OH radicals. It is well known that H_2_O_2_ can trap photoinduced e^-^ to stabilize the paired e^-^ and h^+^. In fact, according to the available literatures, hydrogen peroxide in this study could act as (1) the electron acceptor; (2) the scavenger of positive holes; (c) the scavenger of hydroxyl radicals; or (3) the producer of hydroxyl radicals [58].

#### 3.12.6. Effect of Inorganic Anions

Inorganic anions are of the substances typically seen in real secondary wastewater effluents and notably impact on the catalytic reactions [58]. Figure 8c,d shows the effect of addition of different anions such as Cl^−^, SO_4_^2−^, NO_3_^−^, and CO_3_^2−^ on the photocatalytic degradation of the drug models by the Ag-CuFe_2_O_4_@WO_3_ nanoparticles. The inhibiting effect of the mentioned anions on the photodegradation efficiency of the nanoparticles follows the order of Cl^−^ > SO_4_^2−^ > CO_3_^2−^ > NO_3_^−^. These anions can compete with the drug molecules for the available active sites on the photocatalyst surface, thereby inactivating the photocatalyst [58]. Moreover, the reduction in photodegradation efficiency is attributed to the electrostatic repulsion and the limited active sites in the catalyst [59,60]. In fact, on the catalyst surface these anions act as receptors for ^•^OH radicals, leading to increased electron and hole recombination. The reason for the significant reduction in the photocatalytic degradation efficiency in the presence of CO_3_^2−^ and NO_3_^−^ ions is that these ions act as pH buffers and render the solution alkaline and thereby the surface of the photocatalyst hydroxylated. The as-charged surface repels the drug molecules electrostatically and thus lowers the adsorption capacity of the photocatalyst nanoparticles and their photodegradation efficiency. Furthermore, the free radicals generated at the photocatalyst surface are trapped by CO_3_^2−^ and NO_3_^−^ anions and cannot participate in the degradation process [40].

### 3.13. Stability and Reuse of the Photocatalyst

**Figure 9:**
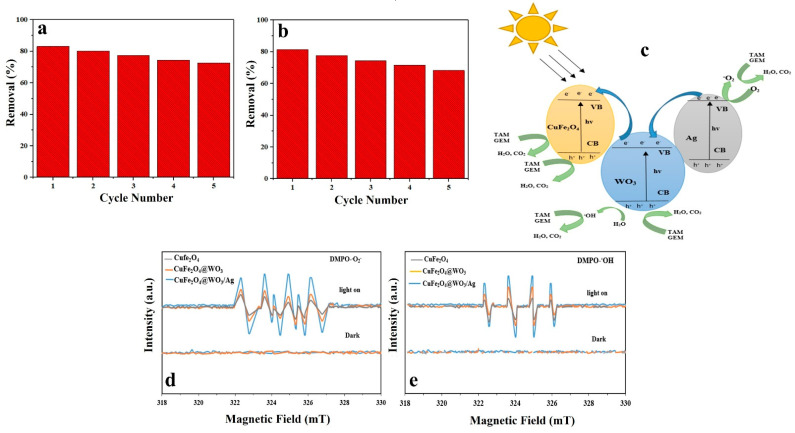
Reusability of the Ag-CuFe_2_O_4_@WO_3_ photocatalyst after five cycles of photodegradation of (**a**) TAM and (**b**) GEM; (**c**) photodegradation pathway of the drug models by the Ag-CuFe_2_O_4_@WO_3_ nanoparticles; ESR spectrum of (**d**) DMPO-◦O_2_ and (**e**) DMPO-◦OH compound for the CuFe_2_O_4_, CuFe_2_O_4_@WO_3_ and Ag-CuFe_2_O_4_@WO_3_ nanoparticles under UV irradiation.

Reusability and stability of a photocatalyst are crucial with respect to its practical and economic application. In this study, reusability of the photocatalyst nanoparticles was challenged in five consecutive cycles. After each cycle, the nanoparticles were separated from the solution using an external magnetic field, washed with distilled water and ethanol, and finally dried in an oven and used for photodegradation of TAM and GEM in a new cycle. As shown in Figure 9a,b, the photocatalytic activity of the nanoparticles did not notably alter after five cycles, implying proper stability and reusability of the Ag-CuFe_2_O_4_@WO_3_ nanoparticles for photodegradation processes. After five cycles, TAM and GEM degradation reduced from 83.15 to 72.64% and from 81.47 to 68.25%, respectively. As indicated the photocatalytic activity loss was insignificant which signifies the photocatalytic stability of these nanoparticles during the photocatalytic reactions. Therefore, not only are the resources saved, but also is the cost of water and wastewater treatment reduced and the process holds great promise for an economic water purification approach. 

### 3.14. Photodegradation Mechanism

The mechanism of photocatalytic degradation of the drugs by the Ag-CuFe_2_O_4_@WO_3_ nanoparticles is demonstrated in Figure 9c. When the nanoparticles are UV irradiated, electrons and holes are generated in the valence band (VB) and the conduction band (CB) of WO_3_, respectively. The photo-excited electrons from the valence band of WO_3_ can recombine with the holes present in the conduction band of CuFe_2_O_4_, and the charge transfer increases the separation of the electron-hole pairs produced in the CuFe_2_O_4_@WO_3_ compound. On the other hand, Fe^+3^ ions act as electron and hole traps, thereby forming Fe^2+^ and Fe^4+^ ions, which are less stable and tend to switch back to Fe^3+^. As a result of this process, active radicals of ^•^OH and ^•^O_2_ are produced [13,39]. Ag nanoparticles are able to accumulate a large density of electrons. In this regard, the photo-excited electrons can be transferred from the CB of WO_3_ to the Ag nanoparticles due to the existing difference between the CB of WO_3_ and the Fermi level of the silver nanoparticles [61]. In other words, due to the capability of Ag nanoparticles to accumulate electrons, the transfer of photoexcited electrons from the WO_3_ surface to Ag leads the overall Fermi surface of the composite nanoparticles to shift to a negative potential. Moreover, since the energy level at WO_3_ is higher than the new equilibrium Fermi surface, the electrons generated in the CB of WO_3_ are transferred to the Ag nanoparticles.

On the whole, Ag nanoparticles act as electron acceptors and thus lower the chance of electron and hole recombination. The photogenerated electrons can be trapped by oxygen and the holes can be trapped by OH. As a result, the highly oxidizing hydroxyl (^•^OH) and superoxide anion radicals (O^◦^_2_^−^) form that can effectively decompose TAM and GEM drug molecules [28]. The main mechanism of the photodegradation process of the drug models can be described by the following reactions:

(8) $$Ag-CuFe_{2}O_{4}@WO_{3}\overset{hv}{\to }e^{-}+h^{+},$$

(9) $$O_{2}+e^{-}\to O_{2}^{{}^{\circ}-},$$

(10) $$O_{2}^{{}^{\circ}-}+2H^{+}\to H_{2}O_{2},$$

(11) $$OH^{-}+h^{+}\to {}^{\circ}OH,$$

(12) $$H_{2}O+h^{+}\to {}^{\circ}OH+H^{+},$$

(13) $${\mathrm{TAM}-\mathrm{GEM}+O}_{2}^{{}^{\circ}-}{+H}_{2}{+\mathrm{OH}+h}^{+}\to \;H_{2}{O+\mathrm{CO}}^{2}{+C}_{3}H_{10}{\mathrm{NO}}_{4}{+C}_{6}H_{14}{O+\mathrm{CH}}_{3}{\mathrm{COOH}+C}_{6}H_{10}O.$$

### 3.15. Electron Paramagnetic Resonance (EPR)

The production of reactive oxidative species (ROSs) by the CuFe_2_O_4_, CuFe_2_O_4_@WO_3_, and Ag-CuFe_2_O_4_@WO_3_ nanoparticles was evaluated through the EPR spin-trap method by DMPO. As shown in Figure 9d, e, no evident signal was recorded in the dark, implying that the removal of TAM and GEM took place solely through adsorption onto the nanoparticles. However, upon UV light irradiation, signals with the intensity of 1:1:1:1 in the DMPO-O_2_ combination appeared that indicated the production of O_2_ radicals. The intensity of this signal was the highest for the Ag-CuFe_2_O_4_@WO_3_ nanoparticles, due to generation of free electrons able to form O_2_ radicals. The EPR signals in the DMPO^− •^OH compound with the intensity ratio of 1:2:2:1 are shown in the Figure 9e. The signals represent generation of oxidative hydroxyl radicals (^•^OH) degrading the TAM and GEM drugs. Therefore, the results of EPR analysis show that ◦O_2_^−^ and ^•^OH are the active species playing role in the photodegradation of TAM and GEM drugs.

### 3.16. Antibacterial Properties

**Figure 10:**
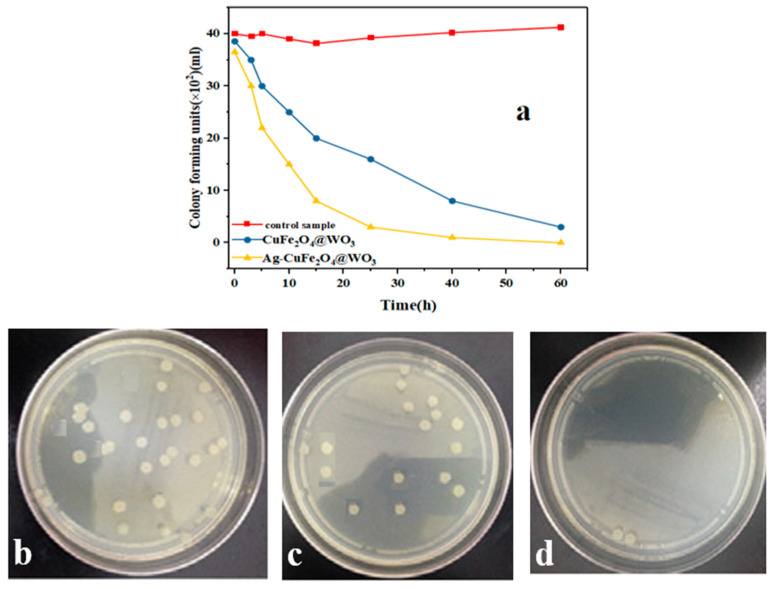
(**a**) Antibacterial properties of the Ag-CuFe_2_O_4_@WO_3_ nanoparticles against *Escherichia coli* over a 60 h incubation period under UV light irradiation. The camera images show the number of the *E. coli* bacteria colonies after 60 h incubation for the petri dishes without the nanoparticles (**b**) and for those containing (**c**) CuFe_2_O_4_@WO_3_ and (**d**) Ag-CuFe_2_O_4_@WO_3_ nanoparticles.

The antibacterial properties of the Ag-CuFe_2_O_4_@WO_3_ nanoparticles are shown in Figure 10a. As seen in this figure, no major change in the concentration of *E. coli* is trackable in the nanoparticle free control sample after 1 h. Meanwhile, this concentration gradually declines for the CuFe_2_O_4_@WO_3_ and Ag-CuFe_2_O_4_@WO_3_ nanoparticle containing media being UV irradiated. Particularly, the highest bactericidal rate was recorded for the Ag doped nanoparticles after 12 h of incubation. Thanks to the presence of Ag, the Ag-CuFe_2_O_4_@WO_3_ nanoparticles show an improved antibacterial efficiency [62]. Ag releases silver ions with well-known bactericidal effect. On the other hand, Ag nanoparticles contribute to further generation of the oxidative radicals that could damage the bacteria membranes. The antibacterial activity of silver nanoparticles on the composite surface can be also associated to their plasmon resonance behavior [63,64]. Figure 10b–d shows that clearly the number of the *E. coli* colonies in adjacent to the CuFe_2_O_4_@WO_3_ and particularly the Ag-CuFe_2_O_4_@WO_3_ nanoparticles declines. At the surface of the CuFe_2_O_4_ and WO_3_ nanoparticles, the electrons injected from Ag are trapped by the O_2_ molecules, and thereby reactive species such as O_2_, OOH◦, HOH are produced that can efficiently kill bacteria [65].

## 4. Conclusions

In this study, we successfully synthesized a nanocomposite nanoparticle photocatalyst system composed of Ag-CuFe_2_O_4_@WO_3_ that could easily be separated and recovered thanks to the presence of CuFe_2_O_4_ magnetic component. Moreover, the system was able to offer a bactericidal effect due to Ag phase. Most importantly, the system could show a superior photodegradation efficiency for the drug pollutants, stemming from a low electron-hole recombination rate realized by the heterostructure of the photocatalyst. The photocatalytic nanoparticles can be reused up to five times with negligible loss of photodegradation efficiency, thus holding promise for a low-cost water decontamination process. Given the high potential of the developed photocatalyst in removal of drug pollutants and bacteria from water, a promising outlook for this system in purification of the multicontaminant water streams is imaginable.

## Data Availability

All data created during this research is openly available from the University of Ardakan Research Data Archive.
